# Evaluation of efficacy and safety for Brucea javanica oil emulsion in the control of the malignant pleural effusions via thoracic perfusion

**DOI:** 10.1186/s12885-018-4328-3

**Published:** 2018-04-12

**Authors:** Dai Fuhong, Gao Xiang, Li Haiying, Wang Jiangye, Gao Xueming, Chai Wenxiao

**Affiliations:** 1 Department of Interventional Medicine, Gansu Provincial Hospital, 204 Dong gang West Road, Lanzhou, 730000 China; 2 First Clinical Medical College, Institute of Hematology, Lanzhou University, Lanzhou, China

**Keywords:** Brucea javanica oil emulsion, BJOE, Malignant pleural effusion, MPE, Meta-analysis, Efficacy, Safety

## Abstract

**Background:**

Brucea javanica oil emulsion (BJOE) is traditional Chinese medicine with implicated anti-tumor activity, which has been used for treating lung cancer in China. The aim of this investigation was to evaluate the effects and safety of intrapleural injection of BJOE in treating malignant pleural effusion (MPE).

**Methods:**

The randomised controlled trials (RCTs) on the effects and safety of BJOE in treating MPE were searched from electronic medical database including MEDLINE, SCI, EMBASE, Cochrance Library and CNKI. A total of 14 RCTs with 1085 patients were involved in this meta-analysis.

**Results:**

The overall response rate (ORR) of traditional chemotherapy drugs plus BJOE was higher than that of traditional chemotherapy drugs alone (*p* = 0.001; odds ratio = 1.39). Meanwhile, the combination of BJOE and traditional chemotherapy drugs improved the quality of life (QOL) of patients with MPE (*p* < 0.001; odds ratio = 1.56) compared with traditional chemotherapy drugs alone. Moreover, the participation of BJOE reduced the myelotoxicity and digestive reactions caused by traditional chemotherapy drugs (*p* < 0.05).

**Conclusions:**

The efficacy and safety of traditional chemotherapy drugs plus BJOE was superior to traditional chemotherapy drugs alone via intrapleural injection in controlling MPE, which suggested that BJOE can be used to treat MPE.

## Background

Malignant pleural effusion (MPE) is a common complication of many malignancies, which denotes an advanced malignant disease process. Most of the MPE are metastatic involvement of the pleura from primary malignancy at lung, breast, and other body sites apart from lymphomas [1]. Clinical practice has found that most lung cancer patients will always be associated with MPE, and lead to lower QOL, and ultimately reduce the life expectancy. Therefore, the treatment of MPE caused more attention of doctors [2]. The present treatments of MPE include the drainage of pleural effusion, intrapleural chemotherapy and systemic chemotherapy. Unfortunately, not all patients with MPE can benefit from quasi chemotherapy and treatment [3]. During the last decade there has been significant progress in unravelling the pathophysiology of MPE, as well as its diagnostics, imaging, and management [4]. Despite its frequent occurrence, current knowledge of MPE remains limited and controversy surrounds almost every aspect in its diagnosis and management [5]. At present, some new drugs studied in China have a certain effect on MPE. These drugs seem to exhibit antitumor activity and low toxicity, they have been used to control MPE [2, 3, 6].

Traditional Chinese Medicines (TCMs) have become increasingly popular in the treatment of cancer in China. Brucea javanica oil emulsion (BJOE) is one of TCMs products, which takes Brucea Jen petroleum ether extracts as raw material and purified soybean lecithin as emulsifier [7]. BJOE (also named yadanzi oil in China) is an extract of the ripe fruit of the simaroubaceae plant Brucea javanica (L.) Merr., which was first recorded in the Supplement to Compendium of Materia Medica. Brucea javanica oil (BJO) contains oleic acid, linoleic acid, stearic acid, palmitic acid, arachidonic acid, and other unsaturated fatty acids [8], which mainly produced in the People’s Republic of China’s coastal tropical and subtropical regions such as Hainan, Guangdong, Guangxi, Yunnan, and other places [9]. The fruit of Brucea javanica has been used for the treatment of various types of cancer in China for centuries. Dozens of single compounds have been isolated and identified from *B. javanica*, which have demonstrated relatively high activities and broad antitumor spectrums in vitro [10]. Previous investigations indicates that BJOE can enhance the chemotherapeutic effect on non-mall cell lung cancer (NSCLC) patients, improve the QOL and reduce adverse effects of platinum-contained chemotherapeutics and thus it is worth referring in clinic [11]. In addition, BJOE combined with chemotherapy could be considered as a safe and effective regimen in treating patients with advanced gastric cancer according to previous study [12].

So far, many investigations have specially disclosed the clinical effectiveness and safety of traditional chemotherapy drugs plus BJOE versus traditional chemotherapy drugs alone in controlling MPE via intrapleural injection. Whether or not BJOE has the potential therapeutic and/or adjuvant therapeutic application in the treatment of human MPE is conflicting. Thus, we performed a systematic literature review to assess the clinical benefit and safety of BJOE combined therapy in controlling MPE.

## Methods

### Identification of literature

We searched and identified relevant RCTs from the databases of MEDLINE/PubMed, EMBASE, Cochrance Library, Web of Science, and CNKI database (from January 2000 to April 2017). The key words applied in the search were as followed: “malignant pleural effusion”, “MPE”, “Brucea javanica oil emulsion”, “BJOE injection”, “BJOEI,” “BJOE,” “Yadanzi”, and “chemotherapy”, “Brucea javanica oil emulsion injection,” “Yadanzi injection,” and “Ya-dan-zi injection.” In addition, if we find that the references of the included studies are closely related to BJOE, we should further search and identify them. The retrieved studies were regarded as potential source and reviewed manually. Moreover, although the published year of these literatures were unlimited, only English and Chinese literatures were involved in this study.

### Data variables of studies

The general data that we selected are as follows: (1) the publication date of each randomized controlled trial; (2) the number of patients included in each study and grouping; (3) the clinical and pathologic features of patients included each study, (4) the patterns of treatment intervention for treating MPE; (5) trials design and implementation. The data on outcomes in present meta-analysis included clinical efficacy, QOL, and adverse effects (AEs) according to World Health Organization (WHO) criteria and Response Evaluation Criteria in Solid Tumors (RECIST). The tumor response included complete response (CR), partial response (PR), stable disease (SD), and progressive disease (PD). The overall response rate (ORR) was defined as CR + PR/overall cases and disease control rate (DCR) was calculated as CR + PR+ SD /overall cases. Toxicity was graded from 0 to IV in severity on the basis of the WHO Recommendations. This meta-analysis only investigated the incidence of Grade II or above.

### Inclusion criteria of the study

Inclusion criteria: (1) study design was confined to RCTs on comparing traditional chemotherapy drugs plus BJOE with chemotherapy drugs alone for treating the MPE; (2) study subjects with MPE must be diagnosed pathologically and (or) cytologically; (3) drugs must be administered by intraluminal injection; (4) outcome measures determined by WHO criteria or RECIST, improvement of QOL evaluated by Karnofsky score (KPS), and AEs assessed by WHO Recommendations for Grading of Acute and Subacute Toxicity must be showed and (6) the sample size of the study must be more than or equal to 60.

### Exclusion criteria of the study

The following criteria were used for the literature exclusion: (1) animal experiments, review, and other irrelevant studies; (2) patient also received other medications; (3) non-RCTs studies; (4) no detailed data about ORR, DCR, evaluation of QOL, and AEs or no indicators for them; (5) investigations were supported by drug producers; (6) lack of comparable control group and (7) single-arm study.

### Supervision of the implementation process

The test design must meet the following rules: (1) RCTs of traditional chemotherapy drugs plus BJOE versus traditional chemotherapy drugs alone via intrapleural injection for controlling MPE; (2) the dosage of BJOE was determined by the suggestions of producers; (3) dosing interval: once a week; (3) number of times of administration: more than or equal to 2 times; and (3) observations on efficacy and safety: ORR, DCR, QOL, and AEs.

### Assessment for quality of RCTs

The criteria of assessment that provided by Cochrane Handbook was employed to evaluate the quality of included investigations. It contained the following Items: (1) sequence generation; (2) how to carry out blinding; (3) how to carry out allocation concealment; (4) how to perform outcome data selective; (5) a description of intention to treat and (6) other sources. According to the above criterion, the quality of trials was defined into three levels: low risk of bias, unclear risk of bias, and high risk of bias [7].

### Statistical methods and analysis

All of the data was calculated by 14.0 (Stata Corporation, TX, USA) software package and Review Manager 5.3 software. The odds ratio (OR) with 95% confidence intervals (CI) was applied to analyze the dichotomous data [6]. By calculating the Z-value of the chi-square test, the statistical *p*-value < 0.05 was considered to be significantly different. The fixed effect model and the random effect model are commonly used statistical models for meta-analysis. According to the presence or absence of heterogeneity, both were selected to measure the safety and efficacy of BJOE pleural perfusion in the treatment of MPE. The χ^2^ statistic and the I^2^ statistic tests were used to assess statistical heterogeneity among included studies [3]. A more common way to indicate the degree of heterogeneity is the statistical test, which is often described as the Cochran chi-square test. A *p* value is often cited as an indicator of the degree of variability in the study. If the *P* value is less than 0.05, no statistical difference is considered, suggesting that the heterogeneity is small. The I^2^ value describes the percentage of variability in point estimates that is due to heterogeneity rather than sampling error, may be readily calculated from most published meta-analyses, and a closed form uncertainty interval is available. If the I^2^ value is less than 50%, the heterogeneity of the study is considered acceptable. If no heterogeneity existed, the method of fixed effects model was adopted, or using the random effects model. To assess the impact of a single study on overall statistical performance, we removed each study from the estimated library one by one, to analyze the impact of each study on overall effectiveness [2]. Further, we employed Begg’s funnel plot and Egger’s test to test the publication bias [7]. The SPSS (version 19.0, Chicago, USA) software was employed to finish the statistics of varying variables. The statistical *p*-value < 0.05 was considered to be significantly different.

## Results

### Literature retrieval process

**Fig. 1:**
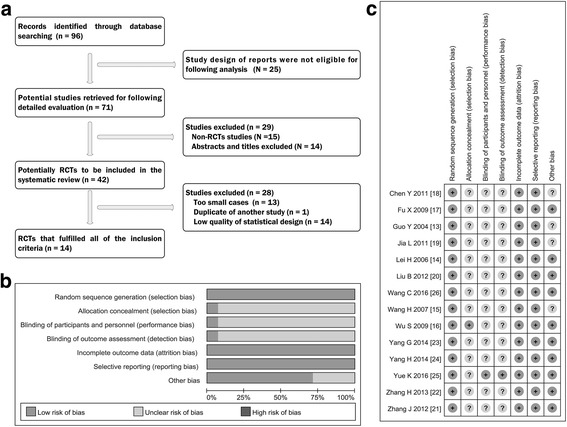
Selection and assessment of literature. **a** Studies were retrieved from the electronic bibliographic databases such as PubMed, Embase, Cochrane Library, Web of Science and CNKI database. **b** and **c** According to the criteria made by the Cochrane Handbook (Version 5.0.1), no heterogeneity existed in eligible RCTs; Overall, these studies had moderate to higher quality

Originally, we conducted the systematic research from online database, and 96 potentially relevant references were yielded. Of them, 25 studies were discarded because the design and implementation of these studies are not eligible for our research analysis. After further screening and eligibility assessment, 29 trials were excluded because some of them were not RCTs and others did not belong to first-hand research data such as summary of meetings, medical reviews and newsletters. Remaining 42 studies seemed to meet the inclusion criterion, but 28 studies were deleted because of the following reasons: repeated data reporting, animal studies, statistical irregularities and too little sample size. Finally, 14 trials were selected as appropriate for inclusion in this meta-analysis. The flow chart showing the selection process was presented in Fig. 1A.

### General characteristics of included studies

The 14 selected trials [13–26] were all RCTs and conducted in China. The qualified 14 studies included a total of 1085 patients, the total number of samples included in the study was from 60 [13, 26] to 123 [16] patients. The volume of pleural effusion of all patients in the amount were all more than 1000 mL and patients’ age varied from 25 [17] to 86 [22] years. From these studies, lung cancer and breast cancer were the most common cause of MPE. A detailed database for meta-analysis on general characteristics was listed in Table 1.

**Table 1:** Data analysis of included studies

Study	N	Male	Female	Age (average)		Histology of Lung cancer				Volume of MPE(N)	Quality of Life	End point
					MPE	LAC	LSCC	SCLC	Others			
Guo Y 2004 [13]	60	40	20	59.5	60	–	–	–	–	> 1000 ml	KPS	RR, DCR, AEs
Lei H 2006 [14]	61	–	–	58–79	61	31		9	–	> 1000 ml	KPS	RR, DCR, SI, AEs
Wang H 2007 [15]	70	45	25	26–81	70	42	7	13	8	Large(46) Moderate(24)	KPS	RR, DCR, SI, AEs
Wu S 2009 [16]	123	86	37	39–75	123	–	–	–	–	> 1000 ml	KPS	RR, DCR, AEs
Fu X 2009 [17]	120	82	38	25–78	120	–	–	–	–	Large(88) Moderate(32)	KPS	RR, DCR, AEs
Chen Y 2011 [18]	61	27	34	43–75	61	–	–	–	–	> 1000 ml	KPS	RR, DCR, AEs
Jia L 2011 [19]	70	38	32	39–85	70	–	–	–	–	> 1000 ml	KPS	RR, DCR, SI, AEs
Liu B 2012 [20]	64	31	33	36–77	64	46	13	5	0	Large(36) Moderate(28)	KPS	RR, DCR, SI, AEs
Zhang J 2012 [21]	64	45	19	28–81	64	20	29	12	3	> 1000 ml	KPS	RR, DCR, SI, AEs
Zhang H 2013 [22]	64	52	12	33–86	64	39	11	14	0	> 1000 ml	KPS	RR, DCR, SI, AEs
Yang G 2014 [23]	94	–	–	–	94	–	–	–	–	> 1000 ml	KPS	RR, DCR, SI, AEs
Yang H 2014 [24]	64	42	22	38–72	64	19	24	13	8	> 1000 ml	KPS	RR, DCR, SI, AEs
Yue K 2016 [25]	111	57	53	–	110	–	70	19	21	> 1000 ml	KPS	RR, DCR, SI, AEs
Wang C 2016 [26]	60	34	26	40–74	60	11	17	–	32	> 1000 ml	KPS	RR, DCR, SI, AEs

*N* number of patients, *MPE* malignant pleural effusion, *LAC* lung adenocarcinoma, *LSCC* lung squamous cell carcinoma, *SCLC* small cell lung cancer, *KPS* karnofsky physical status score, *RR* response rate, *DCR* disease control rate, *SI* symptom improvement, *AEs* adverse effects

### Quality of study design

We found that the number of males (569) was more than the females (546) in the BJOE combined group and control group, respectively. The design of 12 studies were that BJOE combined with cisplatin versus cisplatin alone through thoracic perfusion for treating MPE [13–18, 20, 22–26], one study was BJOE combined with bleomycin versus bleomycin alone [21], another was BJOE combined with oxaliplatin versus oxaliplatin alone [19]. The dosages of BJOE via thoracic perfusion and follow-up times for efficacy evaluation had a good consistency, which was shown in Table 2. All studies had a certain tumor diagnosis by pathology or pleural effusion cytology diagnosis, and KPS score of each patient greater than 50 points. Generally, the dosage of BJOE was administered at the range of 40-100 mg per one time and frequency of administration was two times at least, which were given by thoracic perfusion after drainage of pleural effusions. There was no significant difference between the two groups in the general data (*p* > 0.05), indicating that they had good comparability.

**Table 2:** Assessment method of administration of included studies

Study	Trial group (N)	Control group (N)	Interventions (Groups)		Treatment cycle	Termination of treatment
			Brucea javanica oil emulsion (BJOE) combined with chemotherapeutic agents	Chemotherapeutic agents alone		
Guo Y 2004 [13]	30	30	Cisplatin 150 mg, 1/week BJOE 50 mL, 1/week	Cisplatin 150 mg, 1/W	1 week	> 2 weeks, or pleural effusion disappeared
Lei H 2006 [14]	31	30	Cisplatin 40-60 mg, 1/week BJOE 40-60 mL, 1/week	Cisplatin 40-60 mg, 1/W	1 week	> 1 weeks, or pleural effusion disappeared
Wang H 2007 [15]	35	35	Cisplatin 20-30 mg/m^2^, 1/week BJOE 80-100 mL, 1/week	Cisplatin 20-30 mg/m^2^, 1/5-7D	1/5-7D	> 2 weeks, or pleural effusion disappeared
Wu S 2009 [16]	68	55	Cisplatin 60 mg, 1/week BJOE 50 mL, 1/week	Cisplatin 60 mg, 1/W	1 week	> 4 weeks, or pleural effusion disappeared
Fu X 2009 [17]	60	60	Cisplatin 40/m^2^, 1/week BJOE 100 mL, 1/week	Cisplatin 40/m^2^, 1/W	1 week	> 4 weeks, or pleural effusion disappeared
Chen Y 2011 [18]	31	30	Cisplatin 60 mg, 1/week BJOE 60 mL, 1/week	Cisplatin 60 mg, 1/W	1 week	> 4 weeks, or pleural effusion disappeared
Jia L 2011 [19]	35	35	Oxaliplatin 100/m^2^, 1/week BJOE 60 mL, 1/week	Oxaliplatin 100/m^2^, 1/W	1 week	> 4 weeks, or pleural effusion disappeared
Liu B 2012 [20]	32	32	Cisplatin 40/m^2^, 1/week BJOE 100 mL, 1/week	Cisplatin 40/m^2^, 1/W	1 week	> 4 weeks, or pleural effusion disappeared
Zhang J 2012 [21]	28	36	Bleomycin 45-60 mg, 1/week BJOE 80-100 mL, 1/week	Bleomycin 45-60 mg, 1/W	1 week	> 2 weeks, or pleural effusion disappeared
Zhang H 2013 [22]	34	30	Cisplatin 40-60 mg, 1/week BJOE 40-50 mL, 1/week	Cisplatin 40-60 mg, 1/W	1 week	> 2 weeks, or pleural effusion disappeared
Yang G 2014 [23]	48	46	Cisplatin 40 mg/m^2^, 1/week BJOE 80 mL, 1/week	Cisplatin 40 mg/m^2^, 1/W	1 week	> 3 weeks, or pleural effusion disappeared
Yang H 2014 [24]	32	32	Cisplatin 40 mg/m^2^, 1/week BJOE 50 mL/m^2^, 1/week	Cisplatin 40 mg/m^2^, 1/W	1 week	> 4 weeks, or pleural effusion disappeared
Yue K 2016 [25]	60	50	Cisplatin 40 mg/m^2^, 1/week BJOE 80-100 mL, 1/week	Cisplatin 80 mg/m^2^, 1/W	1 week	> 2 weeks, or pleural effusion disappeared
Wang C 2016 [26]	45	45	Cisplatin 40 mg/m^2^, 1/week BJOE 60 mL, 1/week	Cisplatin 40 mg/m^2^, 1/7d	7D/cycle,2 cycles	> 2 cycles, or pleural effusion disappeared

*BJOE* Brucea javanica oil emulsion, *N* numbers of patients, *D* day, *W* week

### The assessment of heterogeneity

Two investigators of us independently reviewed and assessed the quality of each study according to the criteria shaped by the Cochrane Handbook, which was specialized in evaluating the systematic reviews of Interventions (Version 5.0.1) [3]. As shown in Table 3, we found that 8 of the 14 studies (57.1%) showed the low risk of bias [17, 20–26] and that the remaining 6 investigations [13–16, 18, 19] displayed the unclear risk of bias (42.9%) (Table 3, Fig. 1B, C). We conducted a heterogeneity analysis of included studies. The results showed that chi-squared was 1.61 (Degrees of freedom = 13; *p* = 1.000) and that the value of I-squared (variation in OR attributable to heterogeneity) showed as 0.0%. These results indicated that these included RCTs had very good homogeneity. Combining the clinical information of these studies, we believe that these studies have very good comparability. Based on no heterogeneity, we completed the subsequent statistical analysis using the fixed effects model.

**Table 3:** Design quality of included trials

Study	Region	Sequence generation	Allocation concealment	Blind	Outcome data	Selective outcome reporting	Other sources of bias	ITT	Risk of bias
Guo Y 2004 [13]	Single center	Random number table (SPSS)	Unclear	Unclear	Yes	No	Unclear	Yes	Unclear risk of bias
Lei H 2006 [14]	Single center	Random number table (SPSS)	Unclear	Unclear	Yes	No	Unclear	Yes	Unclear risk of bias
Wang H 2007 [15]	Single center	Random number table (SPSS)	Clear	Unclear	Yes	No	Unclear	Yes	Unclear risk of bias
Wu S 2009 [16]	Single center	Random number table (SPSS)	Unclear	Unclear	Yes	No	Unclear	Yes	Unclear risk of bias
Fu X 2009 [17]	Single center	Random number table (SPSS)	Unclear	Unclear	Yes	No	Clear	Yes	Low risk of bias
Chen Y 2011 [18]	Single center	Random number table (SPSS)	Unclear	Unclear	Yes	No	Unclear	Yes	Unclear risk of bias
Jia L 2011 [19]	Single center	Random number table (SPSS)	Unclear	Unclear	Yes	No	Unclear	Yes	Unclear risk of bias
Liu B 2012 [20]	Single center	Random number table (SPSS)	Unclear	Unclear	Yes	No	Clear	Yes	Low risk of bias
Zhang J 2012 [21]	Single center	Random number table (SPSS)	Unclear	Unclear	Yes	No	Clear	Yes	Low risk of bias
Zhang H 2013 [22]	Single center	Random number table (SPSS)	Unclear	Unclear	Yes	No	Clear	Yes	Low risk of bias
Yang G 2014 [23]	Multiple center	Random number table (SPSS)	Unclear	Unclear	Yes	No	Clear	Yes	Low risk of bias
Yang H 2014 [24]	Single center	Random number table (SPSS)	Unclear	Unclear	Yes	No	Clear	Yes	Low risk of bias
Yue K 2016 [25]	Single center	Random number table (SPSS)	Unclear	Clear	Yes	No	Clear	Yes	Low risk of bias
Wang C 2016 [26]	Single center	Random number table (SAS)	Unclear	Unclear	Yes	No	Clear	Yes	Low risk of bias

*SAS* SAS software, *SPSS* SPSS software, *ITT* intention-to-treat

### Comparison of ORR and DCR between traditional chemotherapy drugs plus BJOE versus traditional chemotherapy drugs alone via intrapleural injection for controlling MPE

**Fig. 2:**
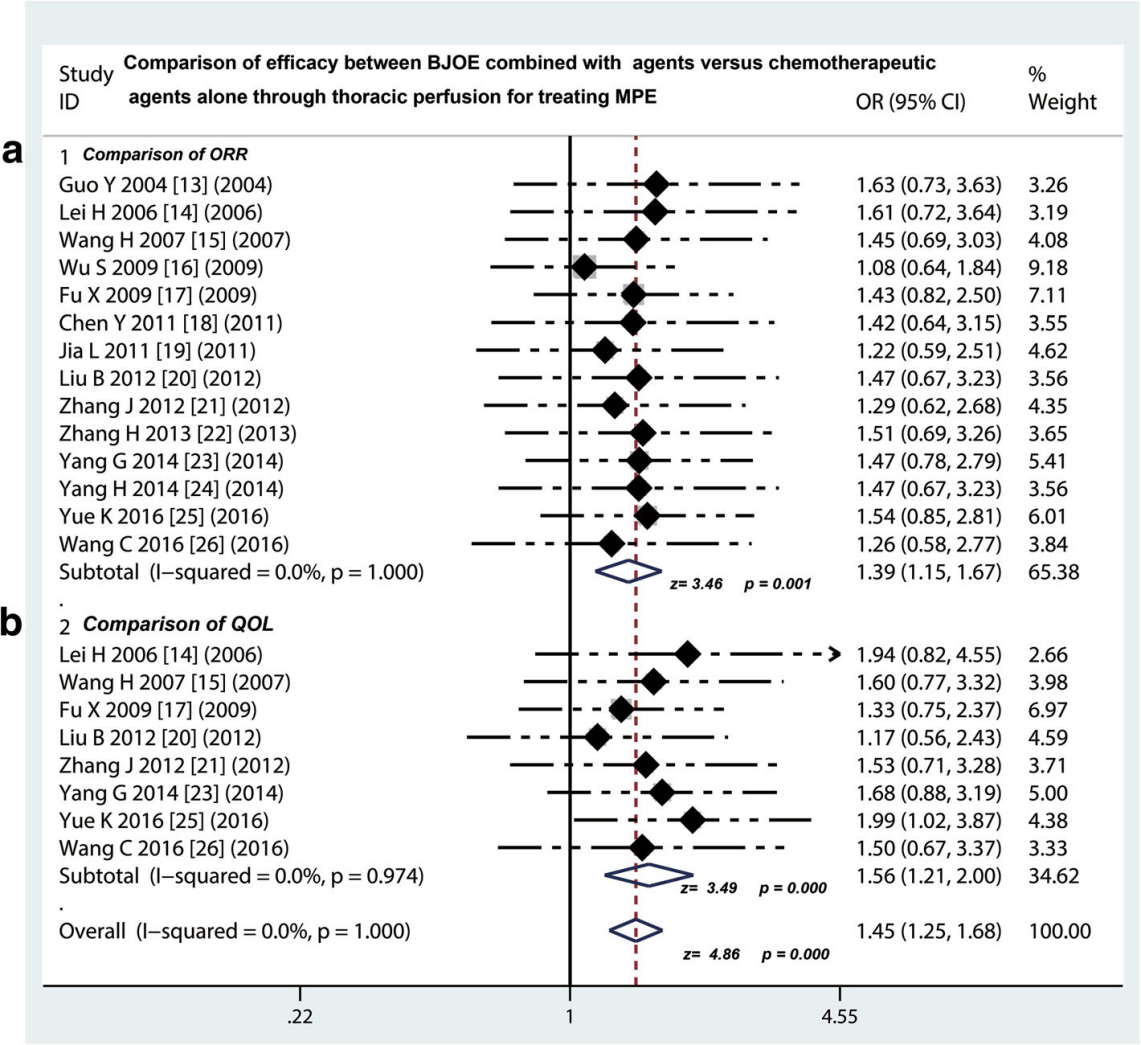
Efficacy comparison of BJOE combined with another agent versus another agent alone by thoracic perfusion for controlling MPE. **a** Thoracic perfusion of BJOE combined with other agents had a higher ORR compared with other agents alone; **b** Thoracic perfusion of BJOE combined with other agents improved the QOL of patients with MPE compared with other agents alone. BJOE, brucea javanica oil emulsion; ORR, overall response rate; MPE, malignant pleural effusion; OR, odds ratio; QOL, quality of life

As shown in Table 4, all of fourteen RCTs [13–26] in this meta-analysis showed the data on comparison of ORR between traditional chemotherapy drugs plus BJOE versus traditional chemotherapy drugs alone via intrapleural injection for controlling MPE. Via the fixed effects model analysis, we found that odds ratio was 1.39 (95% CI 1.15 to 1.67; Z value = 3.46, *p* = 0.001), which suggested that the ORR of traditional chemotherapy drugs plus BJOE was remarkably higher than that of traditional chemotherapy drugs alone (Fig. 2A). In addition, fourteen studies [13–26] showed the data about DCR and displayed that the BJOE combination arms and chemotherapeutic agents single group had the same DCR rate (odds ratio = 1.04, 95% CI 0.888 to 1.23; test for overall effect: Z = 0.44, *p* = 0.663).

**Table 4:** Efficacy of BJOE injection in treating malignant pleural effusion

Study	Study design (N)		Pleural perfusion (N)		Efficacy of therapy									Improvement of SI (N,%)	
			Group 1	Group 2	Group 1					Group 2					
	Group 1	Group 2			CR	PR	SD	PD		CR	PR	SD	PD	Group 1	Group 2
Guo Y 2004 [13]	30	30	BJOE + P	P	11	15	4	0		5	11	14	0	–	–
Lei H 2006 [14]	31	30	BJOE + P	P	11	14	6	0		5	10	15	0	24(77.4)	12(40)
Wang H 2007 [15]	35	35	BJOE + P	P	13	16	6	0		7	13	15	0	32(91.4)	20(57.1)
Wu S 2009 [16]	68	55	BJOE + P	P	39	20	9	0		16	28	11	0	–	–
Fu X 2009 [17]	60	60	BJOE + P	P	22	28	8	2		14	21	18	7	44(73.3)	33(55)
Chen Y 2011 [18]	31	30	BJOE + P	P	12	13	6	0		8	9	13	0	–	–
Jia L 2011 [19]	35	35	BJOE+ L-OHP	L-OHP	10	18	7	0		8	15	12	0	–	–
Liu B 2012 [20]	32	32	BJOE + P	P	18	7	4	3		13	4	9	6	28(75)	24(50)
Zhang J 2012 [21]	28	36	BJOE + BLM	BLM	10	16	2	0		9	17	10	0	25(89.3)	21(58.3)
Zhang H 2013 [22]	34	30	BJOE + P	P	19	10	5	0		11	6	13	0	–	–
Yang G 2014 [23]	48	46	BJOE + P	P	14	26	8	0		4	22	16	4	42(87.5)	24(52.17)
Yang H 2014 [24]	32	32	BJOE + P	P	18	7	4	3		13	4	9	6	–	–
Yue K 2016 [25]	60	50	BJOE + P	P	23	27	10	0		13	14	23	0	43(71.7)	18(36)
Wang C 2016 [26]	30	30	BJOE + P	P	10	14	5	1		8	11	7	4	24(80)	16(53.3)

*N* cases; *Group 1* BJOE combined with chemotherapeutic agents; *Group 2* Chemotherapeutic agents alone, *BJOE* Brucea javanica oil emulsion; *P* cisplatin, *L-OHP* Oxaliplatin, *BLM* bleomycin, *CR* complete response, *PR* partial response, *SD* stable disease, *PD* progressive disease

### Comparison of QOL between traditional chemotherapy drugs plus BJOE versus traditional chemotherapy drugs alone via intrapleural injection for controlling MPE

As shown in Table 4, a total of 8 trials [14, 15, 17, 20, 21, 23, 25, 26] provided the data on comparing the QOL between the BJOE plus traditional chemotherapy drugs versus traditional chemotherapy drugs alone via intrapleural injection for controlling MPE. The QOL improvement was evaluated by the KPS score of patient. After treatment, the KPS score increased by ≥10 points was defined as improvement of QOL. We found that the improvement rate of BJOE combined perfusion group (262/324, 80.86%) was significantly higher than that of chemotherapy group alone (168/319, 52.66%). The results of meta-analysis showed that the odds ratio ranged from 1.17 to 1.99 and the pooled odds ratio in this analysis displayed a value of 1.56 (95% CI 1.21 to 2.00; Z = 3.49, *p* < 0.001), which suggested that BJOE combined with chemotherapeutic agents significantly improved the QOL of patients with MPE, as compared with single chemotherapeutic agents (Fig. 2B).

### Composition ratio of AEs on traditional chemotherapy drugs plus BJOE versus traditional chemotherapy drugs alone via intrapleural injection for controlling MPE

Nine [14, 15, 17, 18, 20, 23–25] of 14 studies compared the AEs on traditional chemotherapy drugs plus BJOE versus traditional chemotherapy drugs alone via intrapleural injection for controlling MPE. As shown in Table 5, the most common AEs in combined group and single group were myelotoxicity (71/363, 19.5% versus 141/350, 40.3%), nausea/vomiting (55/392, 14.03% versus 104/386, 28.4%), liver and renal injury (7/124, 5.6% versus 10/124, 8.1%), chest pain (39/299, 13.04% versus 52/299, 17.39%) and fever (30/309, 9.7% versus 45/305, 14.75%).

**Table 5:** Comparison of adverse events between BJOE combined with chemotherapeutic agents versus chemotherapeutic agents alone

Study	Myelotoxicity (%)		Nausea/vomiting (%)		Liver and renal injury (%)		Chest pain (%)		Fever (%)	
	Group 1	Group 2	Group 1	Group 2	Group 1	Group 2	Group 1	Group 2	Group 1	Group 2
Lei H 2006 [14]	9(29)	22(73.3)	5(16.1)	12(40)			3(9.7)	5(16.7)	3(9.7)	4(13.3)
Wang H 2007 [15]	9(25.7)	21(60)	2(5.7)	5(14.3)	–	–	1(2.9)	1(2.9)	–	–
Fu X 2009 [17]	15(25)	36(60)	4(6.67)	12(20)	1(1.7)	2(3.3)	3(5)	8(13.3)	3(5)	11(18.33)
Chen Y 2011 [18]	9(29.03)	22(73.33)	5(16.13)	12(40)	–	–	3(9.68)	5(16.67)	3(9.68)	4(13.33)
Jia L 2011 [19]	8(22.86)	9(25.7)	15(42.86)	14(40)	–	–	–	–	–	–
Liu B 2012 [20]	5(15.6)	6(18.8)	5(15.6)	7(21.9)	3(9.4)	4(12.5)	3(9.4)	4(12.5)	5(15.6)	4(12.5)
Zhang J 2012 [21]	–	–	2(7.14)	6(16.6)	–	–	0(0)	3(8.3)	3(10.7)	11(30.6)
Zhang H 2013 [22]	–	–	–	–	–	–	12(35.3)	10(33.3)	–	–
Yang G 2014 [23]	8(16.7)	10(26.7)	2(4.17)	6(13.04)	–	–	14(29.17)	16(34.8)	–	–
Yang H 2014 [24]	5(15.6)	6(18.8)	5(15.6)	7(21.9)	3(9.4)	4(12.5)	–	–	5(15.6)	4(12.5)
Yue K 2016 [25]	3 (5.0)	9 (18)	10(16.7)	23 (46)	–	–	–	–	8 (13.3)	7(14)
	*P* < 0.05		*P* < 0.05		*P* > 0.05		*P* > 0.05		*P* > 0.05	

Values are given as number of patients (%).*Group 1* Brucea javanica oil emulsion (BJOE) combined with chemotherapeutic agents; *Group 2* Chemotherapeutic agents alone

### Comparison of AEs between traditional chemotherapy drugs plus BJOE versus traditional chemotherapy drugs alone via intrapleural injection for controlling MPE

**Fig. 3:**
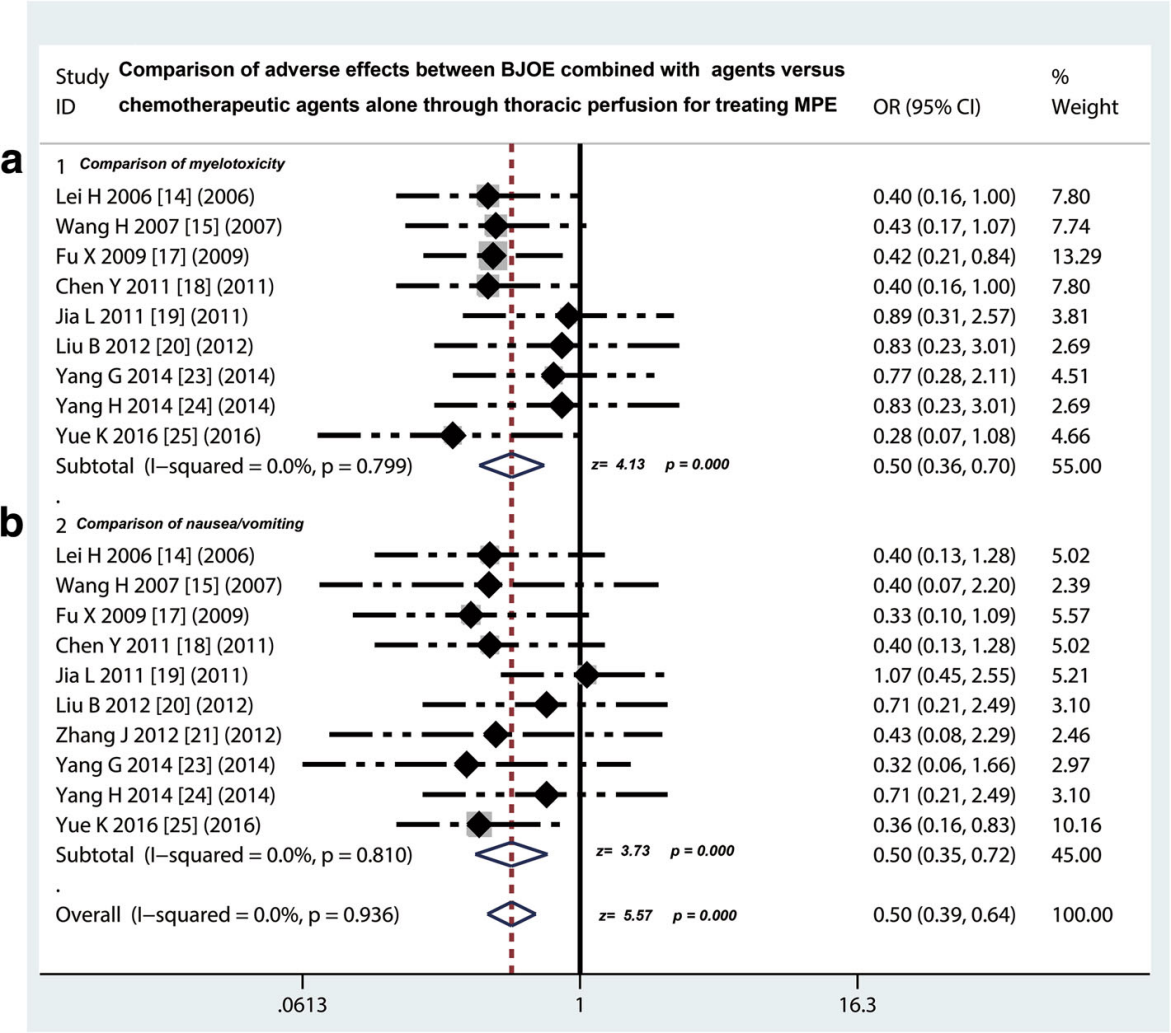
Safety evaluation of BJOE combined with another agent versus another agent alone by thoracic perfusion for controlling MPE. **a** The BJOE combination therapy displayed a lower incidence rate of myelotoxicity than the project of other agents alone; **b** The BJOE combined with other agents had a lower incidence of digestive reactions than and other agents alone. BJOE, brucea javanica oil emulsion; MPE, malignant pleural effusion; OR, odds ratio

**Fig. 4:**
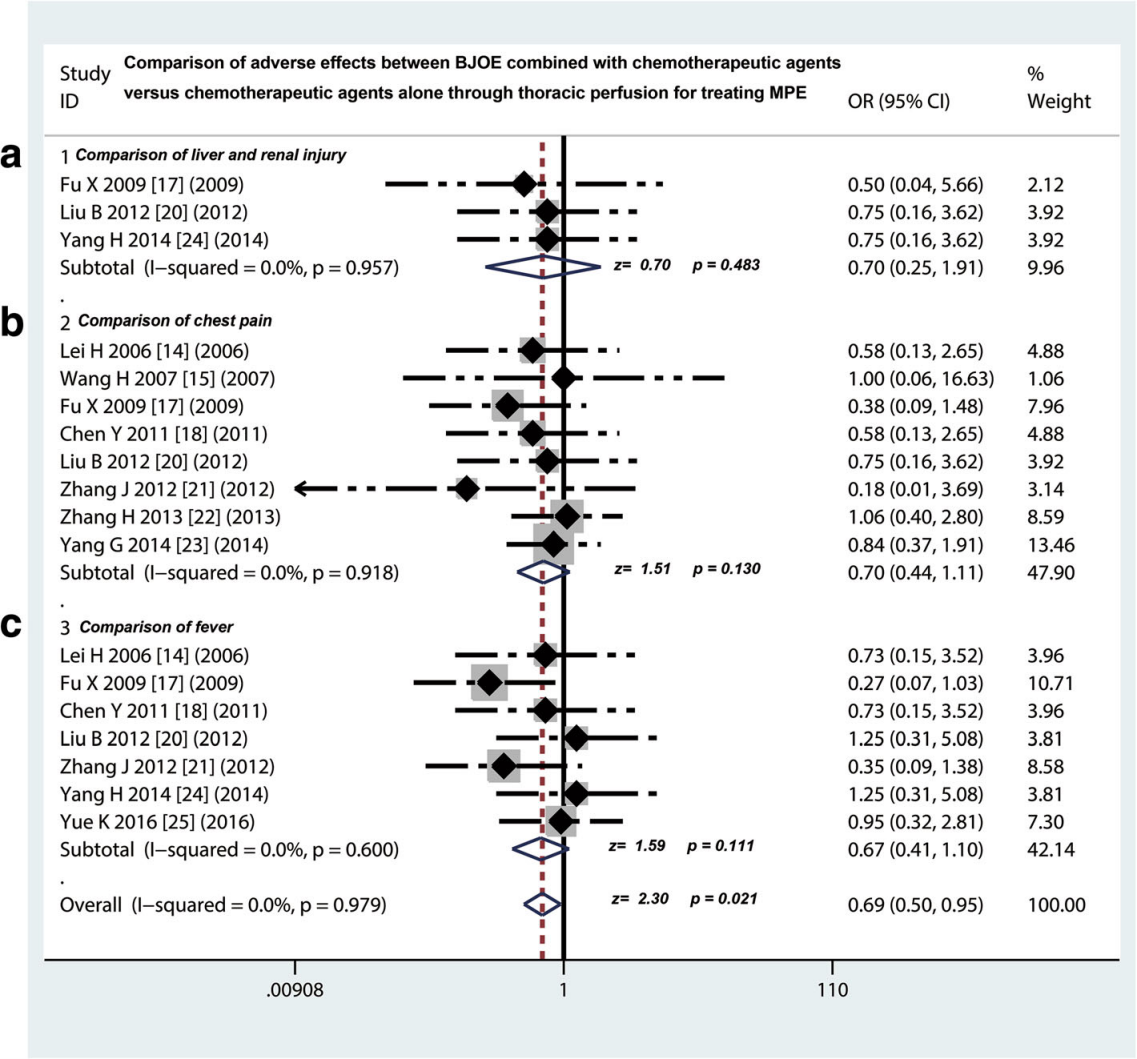
Safety evaluation of BJOE combined with another agent versus another agent alone by thoracic perfusion for treating MPE.**a** No difference in incidence rate of liver and renal injury was testified between BJOE combined with other agents and other agents alone; **b** The incidence of chest pain caused by BJOE combination therapy had the same occurrence probability compared with the other agents alone; **c** The BJOE combined with other agents had the same incidence of fever with other agents alone. BJOE, brucea javanica oil emulsion; MPE, malignant pleural effusion; OR, odds ratio

Nine [14, 15, 17, 18, 20, 23–25] of 14 studie compared the myelotoxicity on traditional chemotherapy drugs plus BJOE versus traditional chemotherapy drugs alone via intrapleural injection for controlling MPE, we found that the incidence rate of myelotoxicity in BJOE combined perfusion group was lower than that of traditional chemotherapy drugs alone group (odds ratio = 0.5, 95% CI 0. 36 to 0.70, *p* < 0.001) (Fig. 3A). Ten [14, 15, 17, 18, 20, 21, 23–25] of 14 studie compared the gastrointestinal reactions, the incidence rate of nausea/vomiting in BJOE combined group also showed a significant decrease compared with traditional chemotherapy drugs alone group (odds ratio = 0.50, 95% CI 0. 35 to 0.72, *p* < 0.001) (Fig. 3B). In addition, three studies compared liver and renal injury (odds ratio = 0.70, 95% CI 0. 25 to 1.91, *p* = 0.483), eight studies compared the incidence of chest pain (odds ratio = 0.70, 95% CI 0. 44 to 1.11, *p* = 0.130), and seven studies compared the incidence of fever (odds ratio = 0.67, 95% CI 0. 41 to 1.10, *p* = 0.111). However, these results suggested that the incidence rate of these AEs did not have differences between both of two projects (*p* > 0.05) (Fig. 4A-C).

### Assessment of publication bias and sensitivity analysis

**Fig. 5:**
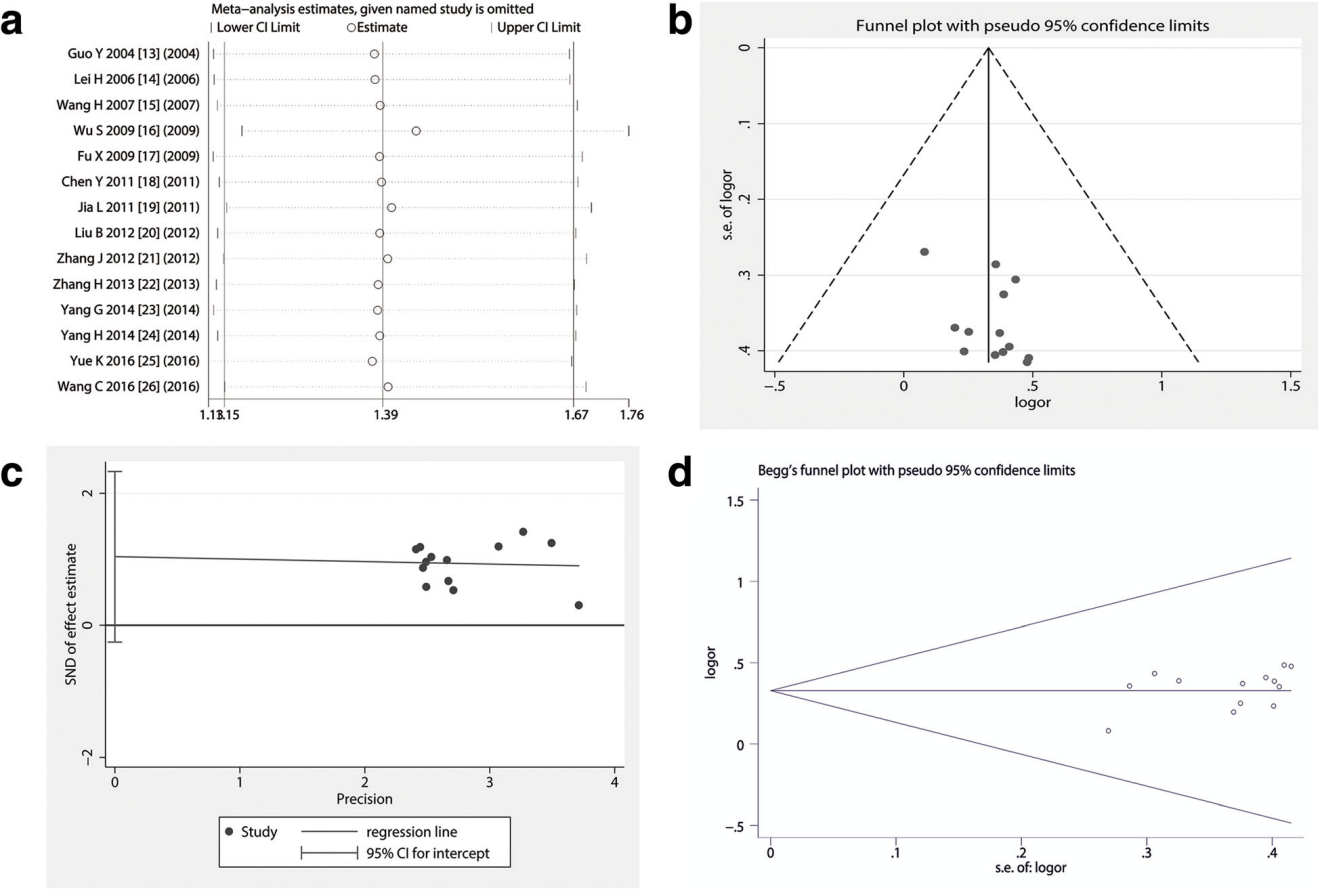
Sensitivity and publication bias analysis.**a** Omitting any trial did not shake the pooled effect of meta-analysis; **b** The shape of the funnel plot appeared to be approximately symmetrical; **c** Egger’s test showed that *p* was 0.105, suggesting included trials did not have a potential impact on the pooled effect of present meta-analysis; **d** Begg’s test showed that *p* value was 0.112, and the funnel plot seems to be nearly symmetrical

Through removing each study, a further meta-analysis was performed to compare with previous results of meta-analysis to explore whether the deleted study have an certain impact on the overall statistical effect [27]. Sensitivity analysis shows that excluding of any study could not change the overall statistical effect, nor could it affect the final statistical conclusion, with an OR pool oscillating between 1.08 and 1.63 (Fig. 5A). We also drew a funnel plot of included studies and noticed that the included studies are symmetrically distributed on both sides of the funnel (Fig. 5B). In addition, for comparing traditional chemotherapy drugs with chemotherapy drugs plus BJOE for controlling MPE, we performed the Egger’s test and the results were as the following: *t* = 1.75 with 13 d.f, *p* = 0.105 (Fig. 5C). We also performed a Begg’s test and the test results showed that Std. Dev. of Score was 18.27, *p* value was 0.112 (Fig. 5D). Come together, the funnel plot, Egger’s tests and the Begg’s test all suggested that publication biases did not have a significant influence on the results.

## Discussion

BJOE is composed of the active ingredients extracted from the ripe fruit of *Sophora flavescens*. The main components are oleic acid and linoleic acid. BJOE is a traditional Chinese medicine. It has been shown that BJOE could directly kill the cancer cells by up-regulating the tumor suppress or genes [7]. Moreover, BJOE has also been found to reverse the tumor cell resistance to chemotherapy and improve the body immunity, without significant AEs [28]. Some experiments show that BJOE is cell cycle non-specific anti-cancer drug, which has an efficacy of killing and inhibition in the G0, G1, S, G2, M phases of tumor cells, and can significantly inhibits DNA synthesis of tumor cells [15, 26, 29]. In addition, previous studies also suggest that the anti-tumor activity of BJOE might be correlated to the mechanism of tumor cell apoptosis, which affects the process of cell cycle, disrupts the cellular energy metabolism, and depresses the expression of vascular endothelial growth factor [7]. So far, a great number of published studies have reported that BJOE can perform a synergetic effect for controlling MPE by improving tumor response and QOL and reducing the incidence of AEs [12–16, 19, 20, 26, 28–30].

We conducted a comprehensive literature search and screening, and finally 14 trials were selected as appropriate for this meta-analysis. By statistical verification and combining the clinical information of these studies, we found that these included RCTs had very good homogeneity and comparability, and further performed a meta-analysis. Our analysis showed that traditional chemotherapy drugs plus BJOE via intrapleural injection had a better ORR benefit compared with traditional chemotherapy drugs alone (odds ratio = 1.39) for controlling MPE, translating into a 22.95% absolute improvement. The results suggested that participation of BJOE exerted an important effect in treating MPE, indicating that BJOE can be used as an alternative drug for controlling the MPE in clinical practice. Previous studies show that the BJOE combination therapy could promote liver cancer cell apoptosis by regulating the expression of soluble Fas/soluble Fas ligand [28] and BJOE also induces apoptosis in the colon cancer cells [28]. Another study finds that BJO-loaded liposomes inhibits the proliferation of hepatocellular cancer HepG2 cells, which appears be dose-dependent, possibly by inducing apoptosis of cancer cells [31]. However, in our study, the BJOE combination seemed to have the same DCR rate (odds ratio = 1.04, *p* = 0.663) compared with chemotherapeutic agents alone. High ORR indicates that the drug can control the disease progress of patients with MPE, meaning that the disease condition of patients was significantly alleviated. At this point, its significance is greater than the control rate because reversing the patient’s disease condition is critical aim of treating malignant tumors [32]. Since the DCR of BJOE combination is comparable to the existing traditional chemotherapy drugs, and it has a high ORR, then the drug should have a certain application value.

Although the control of primary disease is very important, the improvement of QOL in patients is also very critical. Overall survival (OS) has always been considered the “gold standard” for tumor therapy in the study of the therapeutic effects of cancer patients. In today’s clinical trials, the improvement in QOL in patients is increasingly being used to examine efficacy of therapy [32]. Our study showed that presence of BJOE remarkably elevated the QOL of patients with MPE (OR = 1.56, 95% CI 1.21 to 2.0), which responding an absolute 28.2% increase of the QOL, as compared with chemotherapeutic agents alone. That is to say that BJOE-containing therapy improves the ability of QOL of patients with MPE to be about 1.56 times compared with therapy of chemotherapy alone. Previous study points out that BJOE inhibits the proliferation of C6 glioma cells by suppressing the phosphoinositide 3-kinases (PI3K), protein Kinase B (AKT), and nuclear transcription factor-κB (NF-κB) protein expression, which also leads to inhibition of invasiveness of glioma cells, suggesting that the anti-tumor effect of BJOE relates to the inhibition of PI3K/AKT signal pathway [30]. The molecular mechanism that BJOE induces apoptosis of T24 bladder cancer cells may be the activation of caspase apoptotic pathway by upregulation of the expression of caspase-3 and caspase-9 proteins and inhibition of the expression of NF-κB and cyclo-oxyge-nase-2 (COX-2) proteins [29]. A meta-analysis has showed that intravenous therapy of BJOE plus chemoradiotherapy may have positive effects on lung cancer patients in response rate, improvement of QOL, and reducing incidences of some AEs compared with chemoradiotherapy alone. However, the results need to be viewed with caution because of low quality of the included studies [33].

The antineoplastic agent cisplatin is widely used for treating lung cancer as it is highly effective. Unfortunately, the AEs are frequently encountered in platinum-based chemotherapy. With rising cancer survival rates, a greater proportion of patients with cancer are living with the AEs of their chemotherapy treatments. Consequently, the QOL of cancer survivors has now become a major concern for clinicians [34]. In our study, whether BJOE plus traditional chemotherapy drugs or traditional chemotherapy drugs alone via thoracic perfusion, the most common AEs are hematopoietic dysfunction and gastrointestinal symptoms, but most of them are grade 1 and grade 2, and patients are better tolerated. However, we excitedly found that the incidence of myelotoxicity and digestive reactions in treatment of traditional chemotherapy drugs plus BJOE was significantly lower than that in traditional chemotherapy drugs alone, indicating that the BJOE not only exert a impact for treating MPE but also decrease the incidence of myelotoxicity and digestive reactions. Unlike traditional antineoplastic agents, previous studies show that BJOE can not only directly kill cancer cells, but also has enhanced immune function and bone marrow hematopoietic function [7, 11]. Our further analysis found that the incidence rate of liver and renal injury, chest pain and fever of BJOE combination therapy had the same occurrence compared with chemotherapeutic agents alone (*P* > 0.05), suggesting that BJOE participation did not increase the incidence of these AEs. So far, more data have exhibited that the BJOE therapy could be well tolerated and had a better safety for clinical application.

For meta-analysis, heterogeneity testing is important because the heterogeneity of the study will affect the overall statistical effect. In order to insure the comparability, it is necessary to do method comparison and bias evaluation. In the funnel plot analysis of publication biases (the contrast of homozygous genotype plotted against the precision) [35], the shape of the funnel plot appeared to be approximately symmetrical, and the magnitude of the main ORs was in dispersion on the right side of 1. The Egger’s test is based on a linear regression of the standard normal deviate against its precision [35]. In our study, the Egger’s tests and the Begg’s test all suggested that publication biases may not have a significant influence on the results. Sensitivity analysis can estimate the impact of a single study on overall statistical performance. Our study suggested that the included studies had excellent homogeneity and were comparable.

However, we also found some of the defects that existed in the meta-analysis study. First, the vast majority of the samples included in the study were small, thus reducing the test efficiency. Second, included studies in this meta-analysis rarely describes whether implements the allocation hiding, inadequate implementation may exaggerate efficacy. Third, most of patients were from China (because BJOE was approved by the China State Food and Drug Administration), which may lead to geographical and ethnic differences. In spite of this, our results still propose a significant suggestion that the BJOE is effective and safe, and it is an alternative for controlling MPE.

## Conclusion

Intrapleural injection of traditional chemotherapy drugs plus BJOE has a better benefit of ORR for treating MPE and improves the QOL of MPE patients, compared with traditional chemotherapy drugs alone. In addition, the participation of BJOE can reduce the toxicity caused by chemotherapy drugs. However, rigorously RCTs should be needed before it is recommended widely.

## Abbreviations

AEs: Adverse effects
AKT: Protein Kinase B
BJO: Brucea javanica oil
BJOE: Brucea javanica oil emulsion
CI: Confidence intervals
CNKI: China National Knowledge Infrastructure
COX-2: Cyclooxygenase-2
CR: Complete response
DCR: Disease control rate
EMBASE: Excerpt Medica Database
HRQOL: Health-related quality of life
KPS: Karnofsky score
MPE: Malignant pleural effusion
NF-Κb: Nuclear transcription factor-κB
NSCLC: Non-mall cell lung cancer
OR: Odds ratio
ORR: Overall response rate
OS: Overall survival
PD: Progressive disease
PI3K: Phosphoinositide 3-kinase
PR: Partial response
QOL: Quality of life
RCTs: Randomised controlled trials
RECIST: Response evaluation criteria in solid tumors
SD: Stable disease
sFas/sFasL: Soluble Fas/soluble Fas ligand
SFDA: China State Food and Drug Administration
TCMs: Traditional Chinese Medicines
WHO: World Health Organization
Yadanzi: The another name of BJOE in Chinese
